# Discovery of *in vivo* Virulence Genes of Obligatory Intracellular Bacteria by Random Mutagenesis

**DOI:** 10.3389/fcimb.2020.00002

**Published:** 2020-02-04

**Authors:** Hannah Bekebrede, Mingqun Lin, Omid Teymournejad, Yasuko Rikihisa

**Affiliations:** Department of Veterinary Biosciences, College of Veterinary Medicine, The Ohio State University, Columbus, OH, United States

**Keywords:** *Ehrlichia* HF strain, Himar1 transposon mutagenesis, mouse virulence, inflammatory cytokines, staphylococcal superantigen-like domain, obligatory intracellular bacteria

## Abstract

*Ehrlichia* spp. are emerging tick-borne obligatory intracellular bacteria that cause febrile and sometimes fatal diseases with abnormal blood cell counts and signs of hepatitis. *Ehrlichia* HF strain provides an excellent mouse disease model of fatal human ehrlichiosis. We recently obtained and established stable culture of *Ehrlichia* HF strain in DH82 canine macrophage cell line, and obtained its whole genome sequence and annotation. To identify genes required for *in vivo* virulence of *Ehrlichia*, we constructed random insertional HF strain mutants by using Himar1 transposon-based mutagenesis procedure. Of total 158 insertional mutants isolated via antibiotic selection in DH82 cells, 74 insertions were in the coding regions of 55 distinct protein-coding genes, including TRP120 and multi-copy genes, such as *p28/omp-1, virB2*, and *virB6*. Among 84 insertions mapped within the non-coding regions, seven are located in the putative promoter region since they were within 50 bp upstream of the seven distinct genes. Using limited dilution methods, nine stable clonal mutants that had no apparent defect for multiplication in DH82 cells, were obtained. Mouse virulence of seven mutant clones was similar to that of wild-type HF strain, whereas two mutant clones showed significantly retarded growth in blood, livers, and spleens, and the mice inoculated with them lived longer than mice inoculated with wild-type. The two clones contained mutations in genes encoding a conserved hypothetical protein and a staphylococcal superantigen-like domain protein, respectively, and both genes are conserved among *Ehrlichia* spp., but lack homology to other bacterial genes. Inflammatory cytokine mRNA levels in the liver of mice infected with the two mutants were significantly diminished than those infected with HF strain wild-type, except IL-1β and IL-12 p40 in one clone. Thus, we identified two *Ehrlichia* virulence genes responsible for *in vivo* infection, but not for infection and growth in macrophages.

## Importance

Ehrlichiosis, a sometimes deadly febrile disease in man and animal, is caused by infection with Gram-negative obligatory intracellular bacteria, *Ehrlichia. Ehrlichia* species lack typical pathogen-associate molecular patterns (PAMPs), such as lipopolysaccharide, peptidoglycan, pili, and flagella, yet they induce acute and/or chronic inflammatory cytokines in infected animals. Studies to identify virulence factors and PAMPs of *Ehrlichia* species are hampered by the limitation to applicable genetic tools and small laboratory animal models. Mouse infection with *Ehrlichia* HF strain provides an excellent model for fatal human ehrlichiosis, as the HF strain is highly virulent in laboratory mice. However, due to inability to culture the HF stain, the use of this model has been limited. As we have succeeded in culturing and whole genome sequencing *Ehrlichia* HF strain, here, we applied Himar1 transposon random mutagenesis system to this bacterium, and analyzed virulence of the mutant *Ehrlichia* strains in the immunocompetent laboratory mice. The *Ehrlichia* HF strain mutagenesis and mouse model provide insights toward *in vivo* virulence factors and PAMPs of *Ehrlichia* pathogens.

## Introduction

*Ehrlichiae* are obligate intracellular, gram-negative cocci that infect wild and domestic animals, and humans, and cause emerging infectious diseases called ehrlichiosis. Ehrlichiosis is a tick-borne zoonosis, and not directly transmitted between humans and/or animals. Four different species of *Ehrlichia* are known to infect humans: *E. chaffeensis, E. ewingii*, Venezuelan Human Ehrlichiosis (VHE) of *E. canis*, and *E. muris* subsp. *eauclairensis* subsp. nov. (Anderson et al., 1991; Perez et al., 1996; Buller et al., 1999; Pritt et al., 2011, 2017). *E. chaffeensis, E. canis*, and *E. muris* infect monocytes and macrophages, whereas *E. ewingii* infects granulocytes, and they cause subclinical or serious sometimes fatal infectious diseases (Buller et al., 1999; Paddock and Childs, 2003; Pritt et al., 2011). Human ehrlichiosis is characterized by fever, headache, myalgia, thrombocytopenia, leucopenia, and elevated liver enzyme levels (Perez et al., 1996, 2006; Buller et al., 1999; Paddock and Childs, 2003; Martinez et al., 2008; Pritt et al., 2011). Complications, such as pulmonary insufficiency, renal failure, encephalopathy, and disseminated intravascular coagulation can occur in fatal human cases of *E. chaffeensis* infection (Paddock et al., 1997). Human ehrlichiosis have been reported from the United States, Europe, Asia, Africa, and South America, although most of the case reports have originated in the United States. Since many human ehrlichiosis cases are neither reported nor diagnosed, the number of reported cases so far likely significantly underestimates the true incidence of the disease. Despite this, the number of human ehrlichiosis cases reported to CDC in the year 2018 was around 1,525, which showed a 10-fold increase in the incidence of the disease over a 10-year period, with the highest incidence among individuals aged more than 60 years (Centers for Disease Control and Prevention, 2018). Although the infection can be treated with broad-spectrum antibiotic doxycycline, the disease is of particular threat in the immuno-compromised and the elderly, and can result in severe morbidity and mortality if left untreated (Walker and Dumler, 1996; Paddock and Childs, 2003).

No known pathogen-associated molecular patterns (PAMPs) including endotoxin, peptidoglycan, flagella, common pili, or exotoxin has been detected in *Ehrlichia* spp., yet they induce acute and/or chronic inflammatory cytokines production in MyD88-dependent, but Toll-like receptors (TLR)-independent manner (Koh et al., 2010; Miura et al., 2011; Rikihisa, 2015). Several virulence factors of *E. chaffeensis*, which are required for bacterial entry, survival and proliferation of macrophages, have been demonstrated, including ehrlichial invasin that binds host cell receptor and triggers its entry into host cells, three type IV secretion system effectors, three pairs of two-component regulatory system, and outer membrane porins for nutrient acquisition (Kumagai et al., 2006, 2008; Mohan Kumar et al., 2013; Rikihisa, 2015, 2017; Lin et al., 2016; Sharma et al., 2017; Teymournejad et al., 2017; Yan et al., 2018). However, whether *Ehrlichia* spp. have *in vivo* virulence factors which are not related to bacterial entry and growth in macrophages is unknown.

*E. chaffeensis* naturally infects dogs and deer with mild to no clinical signs (Dawson and Ewing, 1992; Davidson et al., 2001; Unver et al., 2002). However, the difficulty, expense, and lack of inbred or gene knockout in these animal models, makes screening *in vivo* virulence factors prohibitive. On the other hand, although *E. chaffeensis* only transiently infects immunocompetent laboratory mice (Ohashi et al., 1998a), *Ehrlichia* sp. HF strain (also called *Ixodes ovatus* Ehrlichia, IOE), which was isolated in Japan from *Ixodes ovatus* ticks, is highly virulent in laboratory mice (Fujita and Watanabe, 1994; Shibata et al., 2000; Okada et al., 2001, 2003). Mice inoculated with HF strain bacteria develop severe clinical signs within 7 days and die within 10 days (Fujita and Watanabe, 1994; Shibata et al., 2000). Thus, mouse infection with HF strain can serve as a model for fatal human ehrlichiosis. The HF strain causes a toxic shock-like syndrome in mice, involving many inflammatory factors mediated by CD8^+^ and CD4^+^ lymphocytes, NKT cells, and neutrophils (Ismail et al., 2004; Stevenson et al., 2008; Yang et al., 2013). The HF strain was not previously culturable, thus all previous studies were performed using the mouse spleen homogenate containing the HF strain (Ismail et al., 2004; Stevenson et al., 2008; Yang et al., 2013). To facilitate studies using the HF strain, we recently stably cultured the HF strain in canine macrophage DH82 cells, and obtained the complete whole genome sequence of the HF strain [GenBank accession NZ_CP007474 (Lin et al., 2013)]. Comparative genome sequence analysis revealed the HF strain is most closely related to *E. muris* subsp. *eauclairensis* subsp. nov., the most recent human ehrlichiosis agent (Pritt et al., 2011, 2017) followed by *E. chaffeensis*.

Obstacles to functional genome analysis of obligate intracellular pathogen are DNA delivery while retaining viability of extracellular bacteria, efficient reintroduction of the transformed bacterial population into host cells, limited selection markers, and the limited efficiency of homologous recombination and transposition systems. Development of the mariner transposase Himar1, which can function in many organisms (Rubin et al., 1999; Pelicic et al., 2000; Ashour and Hondalus, 2003) and the development of hyperactive Himar1 mutants (Lampe et al., 1999) have effectively diminished this last obstacle. Himar transposition provides an ideal system for global genome functional analysis of the AT-rich organism like *Ehrlichia* species (Munderloh et al., 2012; Cheng et al., 2013; McClure et al., 2017), since it recognizes AT sites and inserts at a single site per genome (Lampe et al., 1999). In the present study, we constructed HF strain Himar1 transposon mutant library, and cloned selected mutants and characterized cloned mutant pathogenicity in laboratory mice.

## Results

### Ehrlichia sp. HF Strain Himar1 Insertional Mutant Library

**Figure 1:**
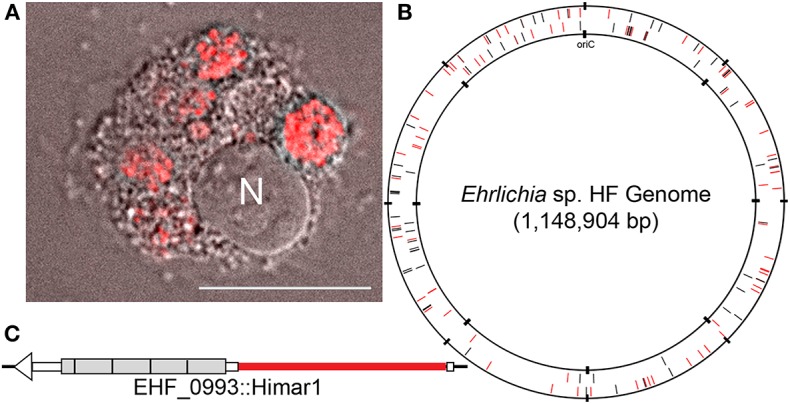
HF mutant and genomic map of the HF strain, showing transposon insertion sites. **(A)** HF mutant H53 expressing mCherry fluorescence in DH82 cells. N, nucleus. mCherry fluorescence/Differential Interference Microscopy. DeltaVision deconvolution microscopy, Scale bar, 10 μm. **(B)** *Ehrlichia* sp. HF chromosomal map showing transposon insertion sites. All mutant bacteria containing the Himar insertions were recovered from DH82 cells by the selection of spectinomycin and streptomycin antibiotics. Black lines represent intragenic insertions and red lines represent intergenic insertions. **(C)** HF mutant H60E (EHF_0993::Himar1). Insertion site is near 5′-end of the gene encoding TRP120 at upstream of the 4 1/4 of the 300-bp tandem repeats (shown in gray boxes) in TRP120, likely disrupting this protein's expression or function.

Host cell-free Ehrlichia sp. HF strain was transformed with the plasmid, pCis-mCherry-SS Himar A7 (Munderloh et al., 2012; Cheng et al., 2013). This plasmid carries a Himar1 mariner transposase, and genes encoding mCherry and downstream spectinomycin/streptomycin antibiotic resistance (aad) that are flanked by the nine base pair inverted repeats recognizable by the transposase with 1,833 base pairs to be inserted into the target genome. Both genes are controlled by the Anaplasma marginale transcriptional regulator 1 (Am-Tr1) promoter, which provides constitutive expression of downstream genes in Ehrlichia organisms for transposition and antibiotic selection. The transformed bacteria were cultured in DH82 cells in the presence of spectinomycin and streptomycin antibiotics to recover mutant bacteria containing Himar insertions. These preparations were observed using Hema3 staining for ehrlichial growth and DeltaVision deconvolution fluorescence microscope for expression of mCherry fluorescence protein in mutant bacteria. Genomic loci of the insertions were identified by semi-random, two-step PCR (ST-PCR) (Chun et al., 1997) using primers listed in Table S1, and mapped to Ehrlichia sp. HF genome by SeqBuilder program in DNAStar Lasergene12 software package. For 23 successful transformation experiments, 158 genomic insertion sites were mapped: among 866 protein-coding genes 74 insertions were present within the coding regions of 55 genes, and the remaining 84 insertions were present within the non-coding regions of the genome (Figure 1, Table 1 and, Table S2). One intergenic insertion site each was mapped to <50 bp upstream of seven distinct genes, and may modify the transcription of these genes (Table S2). In E. chaffeensis Himar1 mutagenesis studies, frequency of mutants is ~5 distinct clones per electroporation (Cheng et al., 2013). In our study, we obtained 1–39 distinct insertional mutations per electroporation. Since the mutants were obtained via antibiotic selection in DH82 cells, mutants in the genes that are essential for survival in the macrophages could not be recovered. All 55 genes that had intragenic insertions have homologs in two human monocytic Ehrlichiosis agents: E. chaffeensis and E. muris subsp. eauclairensis (Table 1 and Table S2, only 34 genes with annotated functions in Ehrlichia sp. HF genome are shown in Table 1, and the remaining 21 genes encode hypothetical proteins without known functions are also included in Table S2). For all 55 genes, E. muris subsp. eauclairensis has the highest % identity among all Ehrlichia species.

**Table 1:** Himar1 insertion sites within genes of assigned functions and % identity of the homologous genes in human pathogens *E. muris* subsp*. eauclairensis* and *E. chaffeensis*.

**Locus ID**	**Genomic insertion site**	**Isolate ID**	**Gene product**	**% aa Identity**	
				***E. muris***	***E. chaffeensis***
EHF_0031	28140	H74C	Chromosome partitioning ATPase ParA	98	95
EHF_0045	43288	H77A-4	Major outer membrane protein OMP1-19	92	84
	43813	H77B-1			
EHF_0048	45484	H82B-2	Major outer membrane protein OMP1-17	93	85
	45586	H75B-1			
	45987	H69F-2			
	46141	H77B-3			
EHF_0066	61587	H67C	Major outer membrane protein OMP1-2	90	65
EHF_0069	64253	H79A-3	Nucleoside diphosphate kinase	96	94
EHF_0075	72763	H73A-1	Dethiobiotin synthase	91	79
EHF_0115	112044	H80B-3	Gamma carbonic anhydrase family protein	92	96
EHF_0125	122477	H81C-2	Peptidase M16 family	93	89
EHF_0135	133819	H43F	Type IV secretion protein VirB2 homolog (VirB2-4)	84	78
EHF_0167	179134	H76F	Aspartate kinase	95	93
EHF_0237	248268	H72E	DNA-3-methyladenine glycosylase	93^a^	87
EHF_0287	308227	H75C	RDD family protein	90	81
EHF_0324	353657	H56B	Lipoate-protein ligase B (LipB)	95	86
EHF_0333	366003	H74B-1	Dihydropteroate synthase	94	56
EHF_0346	382057	H80C-3	Cell division ZapA family protein	96	88
EHF_0382	406316	H55	comEC/Rec2-related domain protein	93	80
EHF_0383	407501	H79E-2	Sodium:alanine symporter family protein	95	90
EHF_0445	490405	H73E-1	Type IV secretion system protein, VirB6 family (VirB6-4)	73^b^	82^b^
EHF_0446	491800	H82C	DUF2460 domain-containing protein	95	86
EHF_0513	566454	H73D-2	Membrane protein, TerC family	90	83
EHF_0515	573935	H81D	Chromosome segregation protein SMC (structural maintenance of chromosomes), archaeal type	55	44
EHF_0522	579965	H69F-1	DNA recombination protein RmuC family protein	94	89
	580467	H34			
EHF_0702	792823	H77A-1	Smr domain-containing protein	87	76
	792889	H67F			
EHF_0703	794748	H53D-2	Thiamine biosynthesis protein ThiC	95	92
EHF_0717	810577	H80D-3	Major Facilitator Superfamily (MFS) transporter	96	83
	810865	H69E			
EHF_0718	813074	H79A-2	Major Facilitator Superfamily (MFS) transporter	94	75
EHF_0723	820620	H80B-4	DNA mismatch repair protein MutS	97	93
EHF_0732	831375	H75F-4	Phage conserved hypothetical BR0599 family protein	90	83
EHF_0743	847362	H76A-1	Glutathione S-transferase family protein	97	94
EHF_0772	892485	H82D-2	DNA mismatch repair protein MutL	93	81
EHF_0775	897331	H81A-2	Putative membrane protein	65	51
EHF_0810	936950	H77F	bolA family protein	83	72
EHF_0877	1016610	H73E-2	Transcriptional repressor NrdR	99	94
EHF_0919	1059627	H72B	Amidophosphoribosyltransferase ComF	93	82
EHF_0956	1098537	H82A-3	Octaprenyl-diphosphate synthase	98	88
EHF_0993	1142873	H60E	120 kDa immunodominant surface protein (TRP120)	38	30

a *Homologous match in E. muris subsp. eauclairensis EMUCRT_RS02460 is a frameshifted pseudogene*.

b *Although E. chaffeensis showed higher % identity compared to E. muris subsp. eauclairensis, it only align with the N-terminal 971 aa of E. chaffeensis VirB6-4 (2,940 aa in total). However, HF strain VirB6-4 was aligned with the full length of E. muris VirB6-4 (1,937 aa), and had higher MAX score (alignment score) (2,763) vs. that of E. chaffeensis (1,789)*.

All Ehrlichia spp. sequenced so far have multiplicated genes encoding immunodominant outer membrane proteins P28/OMP-1 (Ohashi et al., 1998b, 2001; Rikihisa, 2015), some of which were demonstrated to have porin activity in E. chaffeensis (Kumagai et al., 2008), and genes encoding type IV secretion apparatus proteins VirB/D (Rikihisa, 2010, 2017; Gillespie et al., 2016). Of 23 paralogs of p28/omp-1 of the HF strain, insertion was detected in four p28/omp-1s: EHF_0066, EHF_0067, EHF_0045 (two different insertions), and EHF_0048 (four different insertions), showing not all P28/OMP-1s are required for growth in macrophages. There are five virB2 paralogs, and one of which, virB2-4 had insertion. Insertion was also detected in one of four tandem virB6 paralogs, virB6-4. One of two copies of bolA encoding a transcription factor BolA related to stress resistance (Santos et al., 1999; Cheng et al., 2011) had an insertion. In addition to these genes with multiple provisions, a single copy gene trp120, homologous to E. chaffeensis TRP120, a type I secretion system effector, which has been extensively studied (Yu et al., 2000; Luo et al., 2011; Zhu et al., 2011; Dunphy et al., 2013, 2014), had the insertion near 5′-end (Figure 1C), implying this gene is not required for Ehrlichia HF infection of macrophages.

Himar1 intragenic insertion mutants were also identified in genes for vitamin synthesis [thiamine biosynthesis protein ThiC (Lawhorn et al., 2004) and dethiobiotin synthase (Otsuka et al., 1988)], mutation repair [RmuC (Slupska et al., 2000), DNA mismatch repair proteins MutS and MutL (Mansour et al., 2001)], transcriptional regulation [DNA-3-methyladenine glycosylase (Wyatt et al., 1999), transcriptional regulator NrdR (Torrents et al., 2007)], nutrient transport [sodium:alanine symporter family protein (Khani et al., 2018), and two MFS transporters (Quistgaard et al., 2016)], bacterial division and chromosomal segregation [chromosome partitioning ATPase ParA (Lutkenhaus, 2012), cell division ZapA family protein (Small et al., 2007)], and others [amidophosphoribosyltransferase ComF (Bhagavan and Ha, 2015), tellurium resistance protein TerC (Turkovicova et al., 2016), RDD family protein (Shao et al., 2018), glutathione S-transferase (Nebert and Vasiliou, 2004), aspartate kinase (Min et al., 2015), gamma carbonic anhydrase family protein (Hewett-Emmett and Tashian, 1996), octaprenyl-diphosphate synthase (Ashby and Edwards, 1990)] (Table 1). These genes are apparently not required for HF strain infection of macrophages. Six insertions were found within 5% the length of the ORF from the C-terminus of EHF_0075, EHF_0332, EHF_0513, EHF_0717, EHF_0733, EHF_0768; these protein functions may not be disrupted by the Himar1 insert.

### Cloning HF Strain Mutants

**Figure 2:**
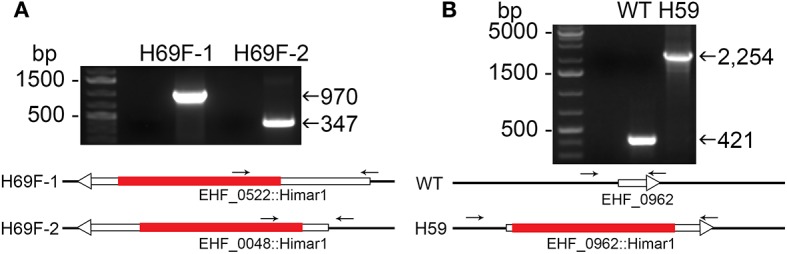
Insertion site-specific PCR to verify insertion site and flank PCR to verify clonality. **(A)** Specific insert PCR showing insertion sites at specific genomic loci of EHF_0522::Himar1 and EHF_0048::Himar1. **(B)** Flank PCR demonstrating clonality of EHF_0962::Himar1, arrows—PCR primers, red bar—Himar1 insert containing aad/mCherry, white boxes with arrow—ORFs. The lengths of ORFs and Himar1 insert were drawn to scale except primers that are enlarged.

**Figure 3:**
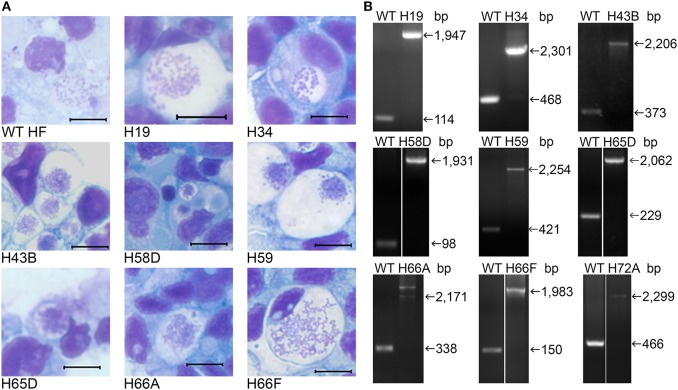
Infectivity of cloned HF Himar1 mutants in ISE6 tick cells. Cloned HF Himar1 mutants and WT HF cultured in ISE6 tick cells. **(A)** HEMA3 staining. Scale bar, 10 μm. **(B)** Insertion site-specific flank PCR confirming clonality of each mutant in tick cells.

Himar1 transposon insertions occur mostly once or rarely twice per genome (Le Breton et al., 2006; Liu et al., 2007; Cartman and Minton, 2010; Cheng et al., 2013). As previous studies with Himar1 transposon mutagenesis of *Anaplasma* and *Ehrlichia* (Felsheim et al., 2006; Cheng et al., 2013), in many rounds of transformation, we obtained a mixture of several insertional mutants, as shown by ST-PCR results from the same cultures. To confirm the presence of Himar1 insertion mutants, insert-specific PCR experiments were performed with primers designed to anneal near identified insertion sites paired with primers annealing to mCherry or *aad* genes in the inserted transposon (Figure 2A and Table S1). To isolate individual clones from the current Himar1 insertion library with 158 genomic insertion sites, limiting dilution of infected DH82 cells was used. To verify the clonality, specific PCR experiments that amplify the genome sequences flanking the insertion site were developed for each mutant clone (Table S1 and Figure 2B). For the flank PCR, primers flanking both sides of the insertion were designed and used to generate PCR products up to 2,500 bp (Figure 2B and Table S1). Wild-type (WT) or other Himar1 insertional mutants, if present, would show the smaller PCR product as they lack the Himar1 insert at this genomic locus. Multiple samples from frozen-thawed cells of 10–20 culture passages (~2 passages/week), were examined by the insertion-specific flank PCR. Of nine clones identified, four were obtained in DH82 cell culture after limiting dilution, and five were clones from original transformation and confirmed by flank PCR (Table 2). Except one clone where the insertion site is at the upstream of type I secretion outer membrane/TolC family protein, the remaining eight clones contain intragenic insertions. All nine clones could infect ISE6 tick cells in culture as shown by HEMA3 staining of infected cells and verified by the flank PCR (Figure 3 and Table 2).

**Table 2:** Characteristics of Cloned HF Himar1 mutants.

**Isolate ID**	**Locus ID**	**Gene product**	**AA**	**Infects ISE6?**	**Infects mice?**	**E. chaffeensis ortholog** **(proteomics coverage)^a^**
H19^b^	EHF_0231	Hypothetical protein	222	Yes	Yes	n/a
H34^b^	EHF_0522	rmuC family protein	433	Yes	Yes	ECH_0577 (0. 584383)
H43B^b^	EHF_ RS04100	Conserved hypothetical protein with SSL domain	651	Yes	Delayed mortality, decreased infection	ECH_0150 (0.383929)
H58D	Intergenic, 184 bp up EHF_0151	Type I secretion outer membrane/TolC family protein	n/a	Yes	Yes	n/a
H59^b^	EHF_0962	Conserved hypothetical protein	119	Yes	Delayed mortality, same infection	ECH_0079 (0.597015)
H65D	EHF_0880	Conserved hypothetical protein	101	Yes	Yes	ECH_0181 (0.252427)
H66A	EHF_0933	Conserved hypothetical protein	84	Yes	Yes	ECH_0130 (0. 357143)
H66F^b^	EHF_0332	Conserved hypothetical protein	353	Yes	Yes	ECH_0379 (0. 45584)
H72A-1	EHF_0150	Conserved hypothetical protein	244	Yes	Yes	ECH_1021 (0.383562)

a *E. chaffeensis Arkansas ortholog protein expression in HL-60 cells (Lin et al., 2011)*.

b *Originally clone*.

### Determination of Mouse LD50 of HF Strain Cultured in DH82 Cells

**Figure 4:**
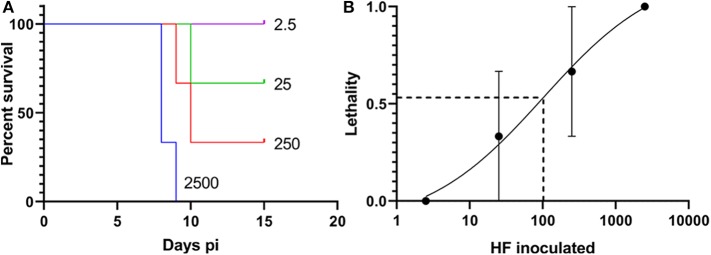
LD50 of the Wild-type (WT) HF strain. **(A)** Kaplan-Meier survival curves for WT HF bacteria, number of inoculated bacteria/mouse is shown for each curve, *N* = 3, Log-rank, Gehan-Breslow-Wilcoxon test among each group: *P* < 0.01. **(B)** Sigmoidal curve showing the calculation of WT HF culture. LD50 is based on survival with 10-fold dilutions, calculated from AAT Bioquest LD50 calculator, with means and standard error (*N* = 3).

The mouse virulence of the HF strain cultured in DH82 cells has not been determined. Therefore, four 10-fold serially diluted WT HF strain (2,500, 250, 25, and 2.5 bacteria) were intraperitoneally inoculated into three female ICR (CD-1) mice each to obtain Kaplan-Meier survival curve. Based on the result, LD50 of HF strain in mice determined by Questgraph LD50 calculator (AAT Bioquest, 2020), was 100 bacteria (Figure 4).

### Mouse Virulence of Cloned HF Mutants

**Figure 5:**
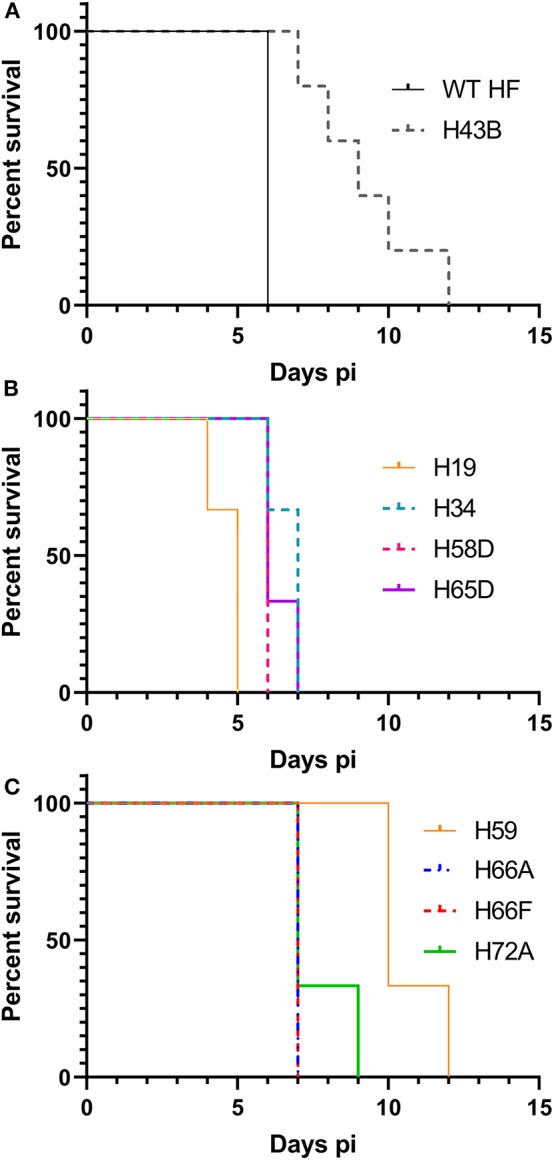
Kaplan-Meier survival curves for mice inoculated with nine cloned mutants. **(A)** Wild type and H43B. Compared with WT, H43B survived significantly longer, *P* < 0.05 by the Log-rank test (*N* = 5). **(B,C)** Compared to H59, each clone *P* < 0.05 by the Log-rank test (*N* = 3). **(B,C)** Were separated for figure clarity.

**Figure 6:**
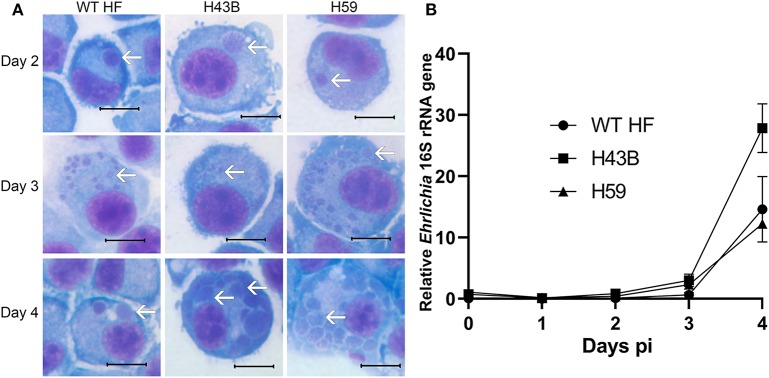
Growth curves of H43B and H59 clones. **(A)** DH82 cells were infected with WT HF, H43B, and H59 clones. Representative HEME3 staining images were shown at day 2, 3, or 4 post-infection. White arrows, ehrlichial microcolonies (morulae). Scale bar, 10 μm. **(B)** Growth curve showing WT HF, H43B, and H59 in DH82 cells as determined by qPCR. Infection and growth of H59 and H43B mutants in DH82 cells were similar by repeated-measures ANOVA, *P* > 0.05 (*N* = 3).

**Figure 7:**
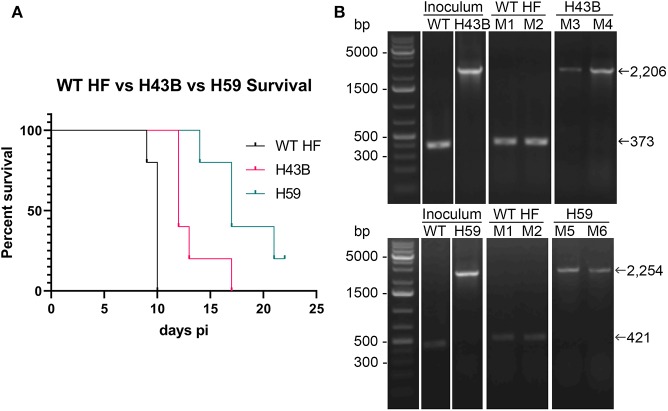
Kaplan-Meier survival curves and flank PCR to detect the clonality of H34B and H59. **(A)** Kaplan-Meier survival curves for two Himar1 mutant clones, *P* < 0.001 by Log-rank, Gehan-Breslow-Wilcoxon test (*N* = 5). **(B)** Flank PCR showing that WT-, H43B-, and H59-infected DH82 cells (inoculum), and liver tissues of Mouse 1 (M1) and Mouse 2 (M2) inoculated with WT and died at day 10 (WT HF), Mouse 3 (M3) and Mouse 4 (M4) died at day 12 and 17, respectively (H43B, top panels), and Mouse 5 (M5) and Mouse 6 (M6) died at day 14 and 21, respectively (H59, bottom panels). For H43B, EHF_RS04100 primers (Table S1) flanking H43B insertion site were used, showing 373 and 2,206 bp without and with Himar1 insert, respectively. For H59, EHF_0962 primers (Table S1) flanking H59 insertion site were used, showing 421 and 2,254 bp without and with Himar1 insert, respectively.

We investigated whether Himar1 insertions affected the HF strain's mouse infectivity and pathogenesis. Immunocompetent mice were inoculated intraperitoneally with nine cloned mutants and WT HF strain at ~5–15 × 10^6^ bacteria/mouse. With this high dosage, most mice became severely moribund and lost >20% body weight and were euthanized, or died at 4–12 days post-inoculation (pi) (Figure 5). However, mice inoculated with H59 and H43B mutants survived significantly longer at 8–12 days pi (Figure 5), hence, these two mutants were selected for further study. H59 has insertion in EHF_0962 (conserved hypothetical protein, 119 aa) and H43B has insertion in EHF_RS04100 (conserved hypothetical protein, 651 aa) (Table 2). Although no known motif was detected in EHF_0962, staphylococcal superantigen-like (SSL) domain (Fraser and Proft, 2008) (aa 429–537, *E*-value 2.47 × e^−04^) was detected in EHF_RS04100. Compared with WT, H59, and H43B infection and growth in DH82 macrophage were similar by quantitative PCR (qPCR) (Figure 6). When ~4 × 10^4^ WT HF were inoculated per mouse, all five mice were severely moribund necessitating euthanasia or died on day 9–10 pi, however mice inoculated with H43B were euthanized or died from day 12 to 17 pi. Mice inoculated with H59 were euthanized or died from day 13 to 21 pi, with one mouse apparently recovering from illness on day 22, when the experiment was terminated (Figure 7A). Clonality of H43B and H59 in inoculated mice were verified by insertion site-specific flank PCR at euthanasia in the liver samples of inoculated mice (Figure 7B). Thus, H43B and particularly H59 mutants have partially reduced virulence in mice.

### Bacterial Load and Cytokine Gene Expression in Mice Inoculated With Cloned Mutants

**Figure 8:**
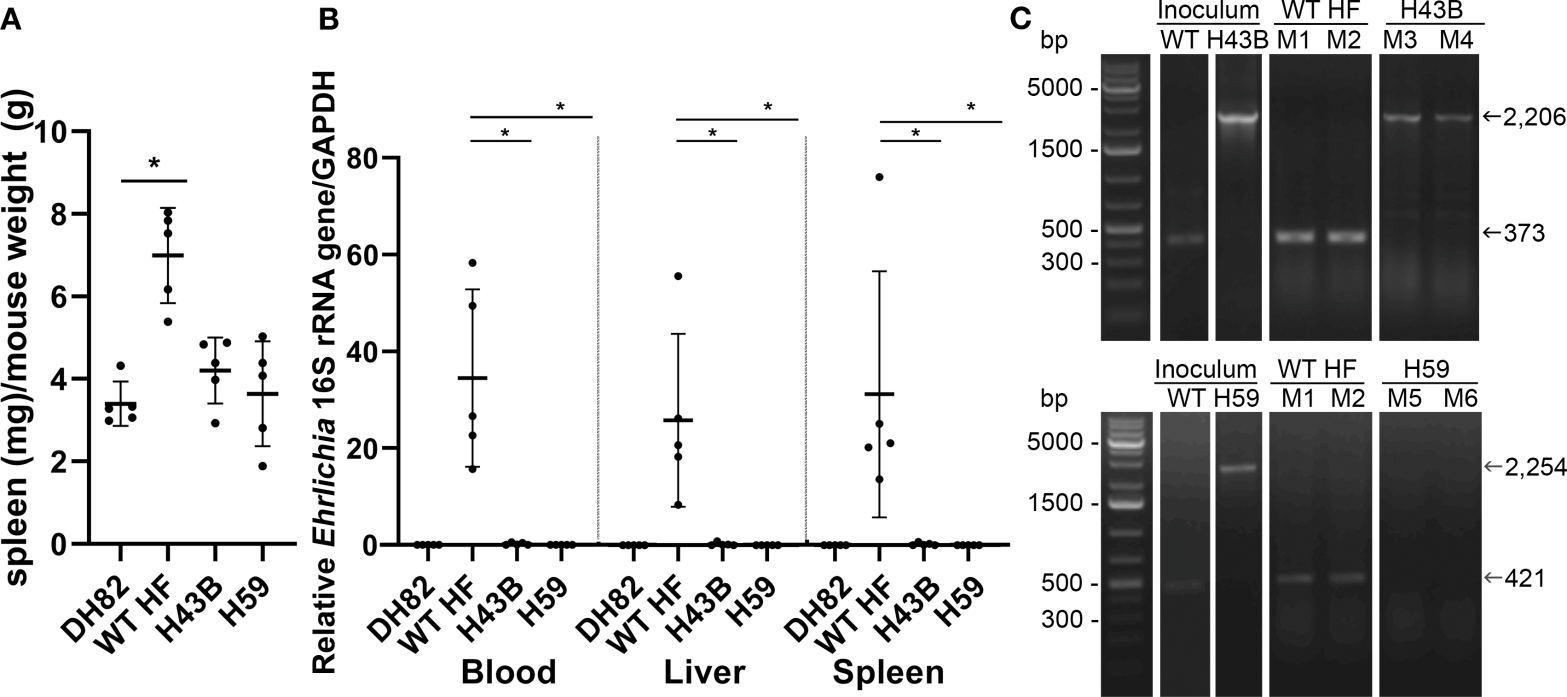
Bacterial load in mice inoculated with H59, H43B, and WT HF strains. **(A)** Spleen index at day 7 pi, *P < 0.05 with ANOVA and Tukey's test (*N* = 5). **(B)** Bacterial load in mouse blood, liver, spleen day 7 pi. 16S rRNA gene was normalized to GAPDH, *P < 0.05 using ANOVA (*N* = 5). **(C)** Flank PCR using primers described in Figure 7B, showing the clonality of H43B- and H59-infected DH82 cells, and in liver tissues of Mouse 3 (M3) and Mouse 4 (M4) for H34B, and Mouse 5 (M5) and Mouse 6 (M6) for H59, respectively at day 7 pi. Mouse 1 (M1) and mouse 2 (M2) were inoculated with WT HF strain, and liver tissues at day 7 pi were used as controls.

**Figure 9:**
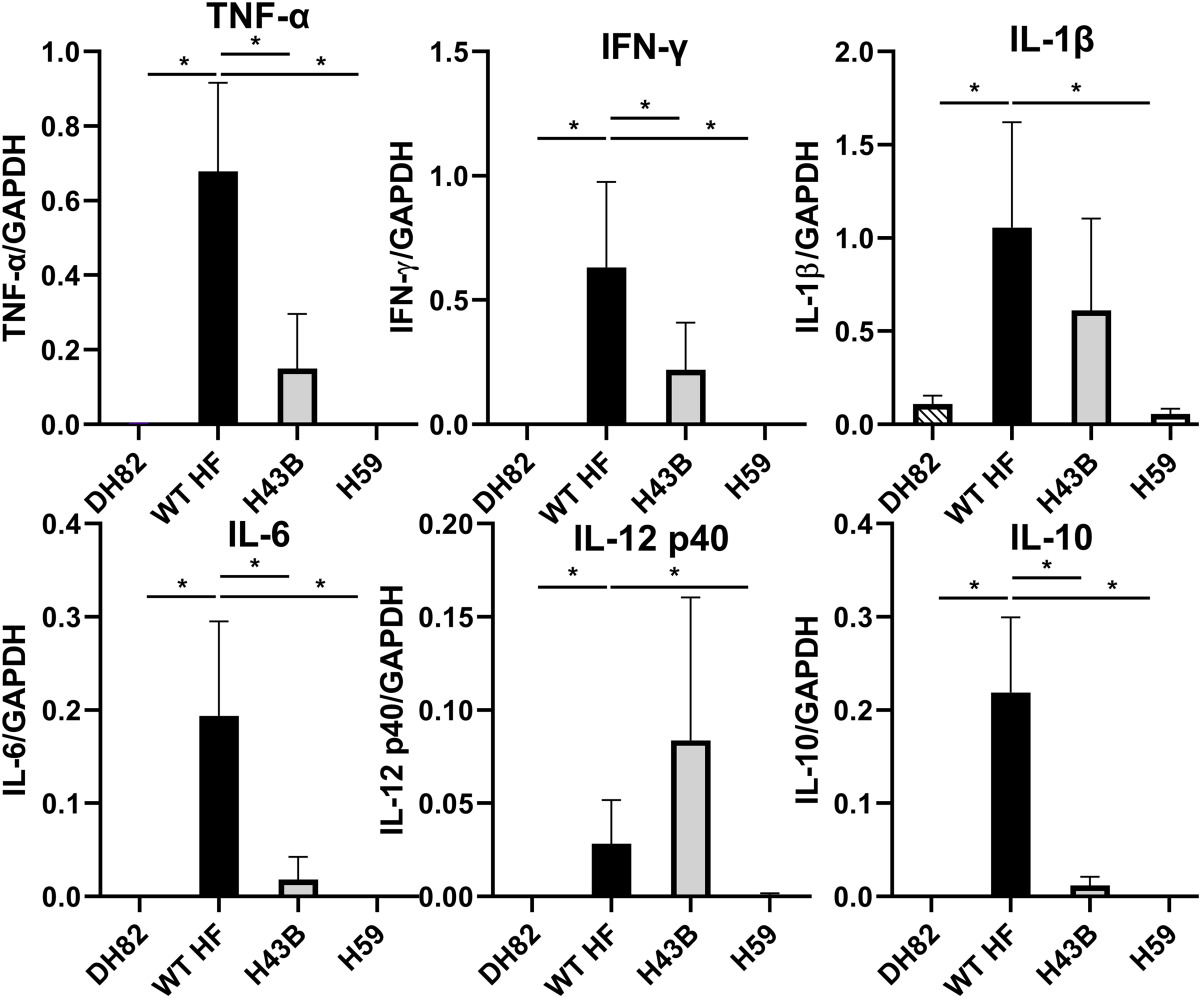
Cytokine gene expression in livers of mice inoculated with H59, H43B, and WT HF strains. qRT-PCR analysis of differentially expressed cytokine genes in livers of mice inoculated with WT and mutant HF strains at day 7 pi. The data shown are means and standard deviations, with mRNA levels normalized by GAPDH, *P < 0.05 using ANOVA followed by Tukey (*N* = 5).

To compare mouse infection and inflammation, mice were inoculated with 600 ~ 3,760 WT, H43B, and H59 bacteria and euthanized at day 7 pi. At day 7 pi, slight splenomegaly was detected with WT, but not with H43B or H59, compared to control mice inoculated with DH82 cells (Figure 8A). In the blood, spleen, and liver, similar levels of bacteria were detected with WT, but dramatically low levels of H43B mutant, and no H59 mutant was detected by qPCR (Figure 8B). Flank PCR showed the clonality of H43B and H59 mutants in DH82 cells prior to mouse inoculation, and the clonality of H43B was confirmed in liver tissues at day 7 pi (Figure 8C). Quantitative reverse transcription PCR (qRT-PCR) results showed that WT HF upregulated proinflammatory cytokines (TNF-α, IL-1β, IFN-γ, IL-6) and immunosuppressive cytokines IL-10 in the liver at day 7 pi (Figure 9). These cytokine mRNAs were undetectable or negligible in the liver of H59-inoculated mice. H43B induced significantly reduced levels of TNF-α, IFN-γ, IL-6, and IL-10 mRNA in the liver compared with WT HF, although IL-1β and IL-12 p40 mRNA levels were similar (Figure 9). H43B mouse inoculation experiment was repeated with ~30-fold more bacteria, but obtained similar results (data not shown).

## Discussion

We have demonstrated that use of the transposon insertional mutagenesis method identified a broad range of 55 ehrlichial genes, which might be unnecessary for this bacterial infection and growth in macrophages. Conservation of these genes among sequenced *Ehrlichia* species suggest that these genes have important roles in the *Ehrlichia* life cycle, other than infecting, surviving, and growing in mammalian leukocytes. Nearly 40% of these genes are predicted to encode hypothetical proteins that lack homology to other bacterial genes, suggesting these genes have evolved to serve unique functions of *Ehrlichia* spp. Among sequenced *Ehrlichia* spp. these genes had the highest homology with genes from *E. muris* subsp. *eauclairensis*, the newest member of human monocytic ehrlichiosis agent discovered in the US. As little is known for pathogenesis of *E. muris* subsp. *eauclairensis*, studies on HF strain will help illuminate this bacterium and its pathogenesis.

Despite reductive genome evolution resulting in a budget genome, P44/Msp2/OMP-1/P28/P30 genes were expanded by duplication in *Ehrlichia* and *Anaplasma* species (Rikihisa, 2010), and genes encoding VirB/D type IV secretion apparatus are expanded among the order Rickettsiales (Rikihisa, 2017), suggesting these duplications are important for the bacterial life cycle. But not all of these genes are required for *in vitro* growth in mammalian cell cultures in the absence of *in vivo* immune pressure. Indeed, our current study and previous publications showed *virB6-4*, the largest and most downstream gene in the *virB6* operon (EHF_0445, APH_0377, MC1_00820) is consistently disrupted by Himar1 transposon without affecting *Ehrlichia, Anaplasma*, or *Rickettsia* growth in mammalian host cells (Felsheim et al., 2006; Lamason et al., 2018). Despite extensive arrays of *in vitro* functions proposed for TRP120 (former gp120) for *E. chaffeensis* infection of macrophages (Yu et al., 2000; Luo et al., 2011; Zhu et al., 2011; Dunphy et al., 2013, 2014), the gene encoding HF strain TRP120 homolog had the insertion near 5′ end, implicating this gene is dispensable for *Ehrlichia* HF infection of macrophages. TRP120 appears to be an abundantly produced protein in *E. chaffeensis* (Yu et al., 2000), TRP mutant of HF obtained in our study would help elucidate *in vivo* or other functions of TRP120 in the future.

The present study found Himar1 insertions in 55 distinct genes. Felsheim et al. (2006) reported isolation of five intragenic insertions APH_0928, APH_0798/0797, APH_0546, APH_0377, and APH_0584 in *Anaplasma phagocytophilum* cultured in HL-60 cells. APH_0584 encodes O-methyltransferase and the mutant is unable to grow in ISE6 tick cells (Oliva Chavez et al., 2015). In the present study, all nine cloned mutants, which were obtained through limited dilutions or antibiotic selection in DH82 cell cultures, could infect ISE6 tick cells. Cheng et al. (2013) identified ECH_0379, ECH_0601, and ECH_0660 intragenic insertions. The mutant of ECH_0660 encoding a phage head-tail connector protein was cloned, which was unable to infect deer, and this mutant was used to vaccinate dogs and protect dogs from *E. chaffeensis* infection by syringe and tick inoculation of *E. chaffeensis* (Nair et al., 2015; McGill et al., 2016). In the present study, ECH_0379 (encoding a conserved hypothetical protein) homolog, EHF_0332 had an intragenic insertion, corroborating that function of this gene is not required for *Ehrlichia* spp. infection of macrophages. Lamason et al. (2018) reported Himar1 insertion in 75 distinct genes of *Rickettsia parkeri* str. Portsmouth (GenBank # NC_017044.1). In the present study, in addition to the VirB6-4 (MC1_00820) ortholog described above, M16 family peptidase (MC1_01650) homolog, EHF_0125, and DNA mismatch repair protein MutS (MC1_02260) homolog, EHF_0723 had insertions, suggesting functions of these genes are universally dispensable for mammalian cell infection by members of the order Rickettsiales. All other mutants were newly detected in the present study. As members of the order Rickettsiales evolutionary share many genes and lifestyle, growing numbers of mutants and their functional characterization, undoubtedly advance our understanding of obligatory intracellular bacteria, their pathogenesis, and adaptation to invertebrate vectors.

There are many virulence factors that were not detected by this method because mutants cannot infect or grow in DH82 cells, or grow too slowly. Of 866 predicted protein-coding open reading frames (ORFs) in the HF genome, over 60% (533) ORFs are house-keeping genes, and knockout mutants of these genes cannot survive in DH82 cell culture systems. Of remaining ORFs, ~60 ORFs of the HF strain are likely required for macrophage infection, as orthologs of *E. chaffeensis* are essential for infection of macrophages including type IV secretion system apparatus (23 ORFs) and effector proteins (3), two-component regulatory system (6), and outer membrane proteins (porin, lipoproteins, invasin; ~30 ORFs) etc. (Kumagai et al., 2006, 2008; Huang et al., 2008; Mohan Kumar et al., 2013; Rikihisa, 2015, 2017; Lin et al., 2016; Sharma et al., 2017; Teymournejad et al., 2017; Yan et al., 2018). Of the remaining ~330 genes, most encode hypothetical proteins or proteins with unknown functions. Our previous RNASeq data (NCBI GEO accession number GPL18497) (Lin et al., 2013) revealed that over 60 ORFs of HF strain were differentially expressed (>2-fold differences, ~70% of which were hypothetical proteins) between ISE6 and canine DH82 cells, suggesting differential requirement of these gene products in two types of host cells. Of 55 ORFs of *Ehrlichia* HF strain with Himar1 insertions that can infect macrophages, six ORFs (EHF_0470, EHF_0051, EHF_0962, EHF_0775, EHF_0242, EHF_0623) were upregulated 2- to 5-fold in the HF strain cultured in ISE6 cells than the HF strain cultured in DH82 cells based on the RNASeq data. Thus, these HF strain genes may function in ticks rather in mammals. Based on these information, we expect maximum 330 knockout intragenic insertional mutants can be recovered by using both tick and mammalian cell lines in the future.

This study identifies two mutants, H43B and H59, which have insertions in a hypothetical protein (EHF_RS04100) and a protein containing staphylococcal superantigen-like domain (EHF_0962), respectively, are required for rapid mouse infection and death. Unlike spotted fever group *Rickettsia* sp. (Lamason et al., 2018), *Ehrlichia* sp. does not produce distinct plaques (host cell lysis), therefore, the limited dilution procedure is required to obtain the clonal population of *Ehrlichia* sp. We established a procedure to verify clonality at every step of cloning and *in vitro* and *in vivo* experiments in this study. We could not, however, deny the possibility that reduced growth of H43B and H59 in mice is due to secondary mutation as complementation experiments are not feasible at this time. However, studies with recombinant proteins, and/or anti-sense protein nucleic acid (Sharma et al., 2017; Yan et al., 2018) will potentially verify these results in the future.

Previously fatal murine ehrlichiosis due to HF strain (derived from infected mouse spleen homogenate) infection is associated with T cell-mediated tissue damage, high levels of serum TNF-α and IL-10, and CD4-Th1 hyporesponsiveness (Stevenson et al., 2008), which are associated with liver pathology and failure to clear HF strain in mice (Ismail et al., 2004). In the present study, we also found cultured WT HF induces strong TNF-α and IL-10 mRNA as well as other proinflammatory cytokines in the liver, which perhaps killed mice; however, reduced levels of these cytokines helped mice inoculated with H43B or H59 lived longer. Although IFN-γ is critical in reducing bacterial growth in macrophages (Barnewall and Rikihisa, 1994), reduced infection of these two mutants in mice did not correlate with IFN-γ mRNA levels. Furthermore, mRNA levels of IL-12 p40 which stimulates NK cells and T helper cells to produce IFN-γ (Hodge et al., 2002) did not correlate with slow growth of mutants in the mice. Interplay of bacteria and host immune system is complex, but liver cytokine response of WT and mutant HF strain suggests hepatitis in ehrlichiosis is related to bacterial load in the liver. Despite of delayed growth of H34B and H59 in mice, they eventually proliferated and killed mice at above certain dosages. *Ehrlichia* HF is, therefore, expected to have additional virulence factors that eventually overcome the mouse immune defense.

Hence, more *Ehrlichia* mouse virulence-related genes are waiting to be discovered. The immunocompetent animal model lends itself to the discovery of genes for *in vivo* virulence but not for infection or growth in macrophage in cell culture, and could be important for differentiating between liver and non-liver virulence mechanisms, which is of major benefit in the absence of accurate *in vitro* measures of the liver and hematopoietic disease caused by *Ehrlichia*. Finally, there are virulence factors that we could not have detected in an animal model. These include factors important for survival in ticks, multiplication within ticks and subsequent spread to humans.

## Materials and Methods

### Ethics Statement

All animal experiments were performed in accordance with the Ohio State University Institutional Animal Care and Use Committee guidelines and approved protocol. The university program has full continued accreditation by the Association for Assessment and Accreditation of Laboratory Animal Care International under 000028, dated 9 June 2000, and has Public Health Services assurance renewal A3261-01, dated 6 February 2019–28 February 2023. The program is licensed by the USDA, number 31-R-014, and is in full compliance with Animal Welfare Regulations.

### *Ehrlichia* sp. HF Culture and Purification

Canine histiocytic leukemia DH82 cells were cultured in DMEM (Dulbecco minimal essential medium; Mediatech, Manassas, VA) supplemented with 5% fetal bovine serum (FBS; Atlanta Biologicals, Lawrenceville, GA) and 2 mM l-glutamine (l-Gln; GIBCO, Waltham, MA) at 37°C under 5% CO_2_ in a humidified atmosphere as described previously (Rikihisa et al., 1991). *Ehrlichia* sp. HF (Shibata et al., 2000) was cultured in DH82 cells with the addition of 0.1 μg/mL cycloheximide (Millipore Sigma, Burlington, MA) at the same condition. The ISE6 cell line, derived from the *Ixodes scapularis* tick embryo, was cultured in L15C300 medium at 34°C as described previously (Munderloh et al., 1999). To assess the degree of bacterial infection in host cells, a drop of infected cells was centrifuged onto a slide in a Shandon Cytospin 4 cytocentrifuge (Thermo Fisher, Waltham, MA), then fixed and stained with HEMA 3 staining solutions (Thermo Fisher).

For transformation, confluent 90% *Ehrlichia*-infected DH82 cells were harvested from three T25 flasks. Host cell–free bacteria were purified by sonication on ice for 8 s twice at output setting 3 using a fine tip on a W380 Sonicator (Heat Systems, Newtown, CT). Lysed cells were centrifuged at 700 × *g* (Sorvall 6000D, Thermo Fisher) to remove unbroken cells and nuclei, filtered sequentially through 5.0- and 2.7-μm nylon syringe filters (Millipore, Billerica, MA), and centrifuged in 40 mL polycarbonate high speed centrifuge tubes (Nalgene Nunc International Corporation, Rochester, NY) at 10,000 × *g* (Sorvall RC 5C Plus using SS-34 rotor) to pellet host cell–free bacteria as described previously (Lin et al., 2016).

### pCis-mCherry-SS-Himar A7 Plasmid Purification and Transformation of *Ehrlichia* sp. HF

pCis-mCherry-SS-Himar A7 plasmid (Cheng et al., 2013) was used for transformation of *Ehrlichia* sp. HF. The plasmids were purified from overnight transformed *dam*^−^*/dcm*^−^ *E. coli* cultures (200 ml) using EndoFree plasmid purification Maxi kit according to the manufacturer's instructions (Qiagen, Germantown, MD). Freshly purified host cell-free bacteria were washed twice with 1 ml ice-cold 0.3 M sucrose and resuspended in 85 μl of 0.3 M sucrose. Bacteria were mixed with 6–8 μg plasmid, transferred to a 2-mm gap electroporation cuvette (Bio-Rad, Hercules, CA), and incubated on ice for 15 min (Yan et al., 2018). Bacteria were electroporated at 2,500 V, 25 μF, and 400 Ω using a Gene Pulser Xcell™ Electroporation System (Bio-Rad). Transformed HF were cultured in 6-well plate with a confluent monolayer of DH82 cells in 1 mL DMEM supplemented with 30% FBS, 4 mM l-Gln, and 10 mM sodium pyruvate (Corning, Corning, NY), incubated at 30°C for 16–20 h to enhance *aad* expression, and then transferred to a 37°C incubator. After 2 days, transformed HF expressing *aad* were selected in the presence of 100 μg/ml spectinomycin, 100 μg/ml streptomycin, and 0.1 μg/ml cycloheximide, and the culture medium containing antibiotics was replaced twice a week until % infected cells reached above 10% (~2 weeks).

### Image Acquisition and Analysis by DeltaVision Microscopy

DH82 cells infected with *Ehrlichia* sp. HF transformed with pCis mCherry-SS Himar A7 plasmids were cytocentrifuged onto a glass slide and fixed in 4% paraformaldehyde (PFA) in phosphate-buffered saline (PBS; 137 mM NaCl, 2.7 mM KCl, 10 mM Na_2_HPO_4_, 2 mM KH_2_PO_4_, pH 7.4) for 20 min at room temperature. After washing the cells with PBS, glass coverslips were mounted on the slide with 70% glycerol mounting medium (Thermo Fisher), and sealed by a nail polish. Fluorescence images with overlay differential interference contrast (DIC) images were captured using a DeltaVision PersonalDV Deconvolution microscope system using TRITC filter sets (GE Healthcare, Marlborough, MA).

### Determine Genomic Insertion Loci Using Semi-random Nested PCR, and Bioinformatics Analysis

The genomic loci of the Himar1 insertion sites were determined by semi-random nested PCR as previously described (Cheng et al., 2013), using primers listed in Table S1. Briefly, DNA was purified from *Ehrlichia* sp. HF mutants using QIAamp DNA blood mini kit according to the manufacturer's instructions (Qiagen). The first-step PCR used transposon-specific outer primers P1 or P5 (Table S1) and a semi-conserved degenerate primer P2, which has a defined adapter sequence at the 5′-end that pairs with the universal primer P4 and a random sequence near the 3′-end to allow for random annealing in *Ehrlichia* chromosome. The first PCR reaction yielded a longer product that served as the template in the second-step PCR reaction using transposon-specific inner primers (P3 or P6) and a second universal primer (P4) that anneals to the 5′-end of primer P2, allowing for specific amplification of the longer PCR product. Residual primers, dNTPs, and enzymes were removed from the first-step PCR products using GeneJET DNA purification kit (Thermo Fisher). The final PCR products were resolved by 1% agarose gel electrophoresis, and the specific DNA bands were purified from the gel using a QIAquick Gel Extraction Kit (Qiagen), and sequenced using transposon-specific primers P3 or P6. The resulting sequences were compared with *Ehrlichia* sp. HF genome sequence (NCBI GenBank accession number: NZ_CP007474.1) by BLASTN (Altschul et al., 1990) to map the transposon insertion sites on the genome. These insertion loci were mapped to the circular *Ehrlichia* sp. HF genome using GenomeViz software (Ghai et al., 2004).

NCBI conserved domain search was used to identify potential function of targeted proteins, and NCBI BLASTP was used to identify homology within the family Anaplasmataceae and other related bacteria. SignalP was used to predict signal peptides and membrane localization (Bendtsen et al., 2004).

### Cloning Mutants and Clonality Confirmation

DH82 cells suspended at 10^4^ cells in 50 μL DMEM culture medium (supplemented with 5% FBS, 2 mM l-Gln, and 0.1 μg/mL cycloheximide) were seeded into each wells of a 96 well flat-bottomed plate (Greiner, Monroe, NC). Approximately 3 *Ehrlichia* sp. HF mutant-infected DH82 cells suspended in 50 μL culture medium were inoculated into each well. After overnight incubation, 100 μL of additional culture medium was added to each well and cells were allowed to grow to confluency. Then monolayers were scraped with sterile pipette tips and ~10^4^ cells in 20 μL were transferred to a new 96-well flat, clear-bottom black plate (Tecan, Morrisville, NC). After cells reached confluency at ~4 days, mCherry fluorescence was detected using a Tecan Infinite M Nano^+^ microplate reader (Tecan). Positive wells were expanded and checked for single insert clones using PCR analysis with primers flanking specifically to the target insertion sites (Table S1). Primers were designed using the NCBI primer-BLAST (Ye et al., 2012).

### DNA Purification and Quantification of *Ehrlichia* Bacterial Numbers

DNA was purified from uninfected and *Ehrlichia* sp. HF-infected DH82 cells and mouse tissues with a QIAamp DNA Mini Kit (Qiagen). To quantify *Ehrlichia*, an absolute quantification method was used by creating a standard curve of the *Ehrlichia* 16S rDNA cloned into a pUC19 plasmid as a standard template (Teymournejad et al., 2017), and bacterial numbers were determined by 16S rDNA copy numbers by qPCR analysis according to the manufacturer's protocol (Stratagene, Waltham, MA) (Liu et al., 2012). The qPCR mixture (20 μl) included 0.25 μM each primer, and 10 μl of SYBR green qPCR master mix (Thermo Fisher). Primer sequences for the *Ehrlichia* 16S rRNA gene are shown in Table S1. PCR was performed in the Mx3000P instrument (Stratagene). The copy number of the targeted gene in DNA samples was calculated by comparing the *Ct* value with the standard curve. The value was normalized against mouse GAPDH levels using specific primers (Table S1).

### qRT-PCR Analysis

WT and mutant-infected mouse liver were preserved in 0.5 cm thickness in RNA*later* reagent (Thermo Fisher). Total cellular RNA was extracted from 30 mg tissue per sample using RNeasy kit (Qiagen) according to the manufacturer's instructions. RNA concentrations and quality were determined by NanoDrop (Thermo Fisher). Total RNA (0.5 μg) was reverse transcribed using a Maxima H Minus First Strand cDNA Synthesis Kit and oligo dT primers (Thermo Fisher). The qPCR reaction mixture (20 μl) included 5 μl cDNA (corresponding to 0.1 μg of total RNA), 0.25 μM each primer, and 10 μl SYBR Green qPCR master mix (Thermo Fisher). PCR was performed in an Mx3000P instrument. Primer sequences are described in Table S1.

### *In vitro* Growth Curve

To generate growth curves of WT, H43B, and H59 mutants of *Ehrlichia* sp. HF in DH82 cell cultures, bacteria were isolated by sonication without filtration as described above, and centrifuged at 10,000 × g for 5 min at 4°C. Supernatant was discarded, and host cell-free bacteria were resuspended in DMEM supplemented with 5% FBS, 2 mM l-Gln, and 0.1 μg/mL cycloheximide, and inoculated on DH82 monolayers in 5 wells/group of 12-well plates. Monolayers were washed three times with media at 2 h pi to remove unbound bacteria. To assess bacterial growth, monolayers were washed three times with PBS, and cells were scraped off in 1 ml PBS. DNA was extracted at each day pi for 4 days, and bacteria numbers were determined by qPCR and normalized by total DNA amount, which corresponds to total cell numbers. One sample of WT and mutant cultures were evaluated by Hema3 staining each day (Oliva Chavez et al., 2015).

### Mouse Infection

In each experiment, infected DH82 cells were resuspended in DMEM medium without FBS and antibiotics, and 0.5 mL per mouse were intraperitoneally inoculated using a 1 mL TB syringe with a 26-gauge needle (BD, Franklin Lakes, NJ). Mice were monitored daily for weight loss and moribund condition indicated by hunched posture, squinted eyes, slow movement, reduced response to stimulation, and loss of 20% body weight according to IACUC. Moribund mice were euthanized by CO_2_ inhalation and cervical dislocation. As bacteria need to be inoculated into mice immediately after harvesting, bacterial numbers were first estimated by counting percent infected cells and approximate numbers of bacteria per cell, and verified by qPCR later as described above. Four sets of experiments were carried out. (1) For LD_50_ determination, female ICR (CD-1) at 4 weeks old (Envigo, Indianapolis, IN) were intraperitoneally inoculated in groups of three with serially diluted 100, 10, 1, and 0.1 DH82 cells containing ~2,500, 250, 25, and 2.5 bacteria, respectively. (2) For screening mutants that lost virulence, male and female C57BL/6 mice at 8–20 weeks old (Envigo) in groups of three to five mice each were intraperitoneally inoculated with 5–15 × 10^6^ *Ehrlichia* sp. HF mutant bacteria from infected DH82 cells. (3) For analysis of mortality of mice infected with H43B and H59, female ICR mice at 10 weeks old (Envigo) in groups of five mice each were intraperitoneally inoculated with 200 infected DH82 cells containing 42,500 WT, 28,400 H43B, or 42,700 H59 bacteria, respectively. Tissue samples were frozen for use in DNA extraction. (4) For H43B and H59 bacterial burden and cytokine analysis, female ICR mice at 6 weeks old (Envigo) in groups of five mice each were intraperitoneally inoculated with 200 infected cells containing 1,330 WT, 600 H43B, and 3,760 H59 bacteria. Mice were euthanized on day 7 pi. Cardiac puncture blood samples were collected and tissue samples were stored in RNALater (Qiagen) for RNA or DNA extraction.

### Statistical Analysis

Statistical analysis was performed with analysis of variance (ANOVA) followed by Tukey multiple comparison, and with repeated measures analysis of variance (rANOVA). *P* < 0.05 was considered statistically significant. Kaplan-Meier survival curves were analyzed by Log-rank, Gehan-Breslow-Wilcoxon test or the Log-rank test. All statistical analyses were performed using Prism 8 software (GraphPad, La Jolla, CA).

## Data Availability Statement

Genome sequences for *Ehrlichia* sp. HF (GenBank accession NZ_CP007474) and transcriptome data (GEO accession number GPL18497) were deposited at NCBI.

## Ethics Statement

The animal study was reviewed and approved by the Ohio State University Institutional Animal Care and Use Committee.

## Author Contributions

YR and ML contributed the conception and design of the study. HB, ML, and OT performed the experiments and analysis. YR, ML, and HB wrote the manuscript. All authors read and approved the submitted version.

### Conflict of Interest

The authors declare that the research was conducted in the absence of any commercial or financial relationships that could be construed as a potential conflict of interest.
