# Supplementary material for: Discovery of in vivo Virulence Genes of Obligatory Intracellular Bacteria by Random Mutagenesis
Source: Front Cell Infect Microbiol. 2020 Feb 4;10:2. doi: 10.3389/fcimb.2020.00002 (PMC7010607; doi:10.3389/fcimb.2020.00002)
Supplement: Supplementary file 1 [file Table_1.pdf]

SUPPLEMENTARY TABLES

Table S1. Primers used in this study *

ST-PCR Primers		
**Primer**	**Primer sequence**	**Target site**
P1 [1]	CTCCATCTTTCAGCTTCAGGCG	mCherry
P2 [1]	GGCCACGCGTCGACTAGTACNNNNNNNNNNGATAT	Degenerate primer
P5	AGTCACCATTGTTGTGCACGACGAC	*aad*
P4 [1]	GGCCACGCGTCGACTAGTAC	Defined primer for P2 nesting
P6	AACCGGCAAAATCGCGCCGAA	*aad* nesting
P3 [1]	GTCTGTGTGCCTTCATACGGTC	mCherry nesting
**Screening primers**		
Himar1	F: ATCAAGCCTTACGGTCACCG<br>R: ATGGGAACGCGTCATGAACT	*aad* and mCherry span

Gene Specific Primers	Locus ID	Product size (bp)
F: TGGTCTAATTATAGTGATGCTATGG<br>R: AGTTTCTTTCTAAAACCATTACG	EHF_0231	114
F: ATGGCATCGGGTTGTTGTTTC<br>R: GACTTTACACAATACGCTTGACTT	EHF_0522	468
F: TCCTCCCAGAATTTTCGCACA<br>R: GAATGTTGCTTTAATTCACCAGC	EHF_RS04100	373
F: CACAAAGAAAGCATATAGTGGAGGT<br>R: ACGCATGTATCGTAAATGCATACT	Up EHF_0151	98
F: TCAATATAGAACAACATACTACAAA<br>R: TCTGCTTCATCAGATTCTACT	EHF_0962	421
F: GCACTGATATTATTTGCAGGTACAG<br>R: GCTTTCCTTGTGTTTTGGCTTCAA	EHF_0880	229
F: TCTACTGATTTGTAAACAATGGAT<br>R: TGCAAGTAGTGCTCCTGTTA	EHF_0933	338
F: ACTGCTATAGCTCTTACATTAGTCT<br>R: GGCATAACCATTTACGAAACACA	EHF_0332	150
F: GGATGAAACTGGTAGGCAACTGATA<br>R: AATAGAGCAATCAACCCAACGGCT	EHF_0150	466
**Insertion Specific Primers**		
F: P3<br>R: TTCCTGACCATTTTGTCTTGCCCA	EHF_0382	549

* Unless cited with a reference, all other primers were designed in this study.

F: P6<br>R: ACCGGAACTTCCTGCTGCAACG	EHF_0135	427
F: TCCTCCCAGAATTTTCGCACA<br>R: P3	EHF_RS04100	588
F: P6<br>R: TAGTTTTGTAAGCTGTGCCCCT	EHF_0733	329
F: TCAGAGCAACAACAGCTGCAATCC<br>R: P3	EHF_0758	746
F: ACGCATGTATCGTAAATGCATACT<br>R: P3	EHF_0151	416
F: P6<br>R: GACTTTACACAATACGCTTGACTT	EHF_0522	970
F: P6<br>R: ACCTGGAGTGTCATTCTCTGATCC	EHF_0048	347

**qPCR primers**		**Purpose**
Mouse GAPDH [2]	F: GTTGTCTCCTGCGACTTCA<br>R: GGTGGTCCAGGGTTTCTTA	GAPDH for normalizing infected mouse tissue
Ehrlichia 16S rRNA [3]	F: CGGGGGAAAGATTTATCGCTATTA<br>R: CGCTTGCCCCCTCCGTATTA	Quantifying *Ehrlichia*
**qRT-PCR primers [4]**		
TNF-α	F: CATCTTCTCAAAATTCGAGTGACAA<br>R: TGGGAGTAGACAAGGTACAACCC	TNF-α expression
IL-1β	F: GGGCCTCAAAGGAAAGAATC<br>R: TACCAGTTGGGGAACTCTGC	IL-1β expression
IFN-γ	F: GCGTCATTGAATCACACCTG<br>R: TGAGCTCATTGAATGCTTGG	IFN-γ expression
IL-6	F: GAGGATACCACTCCCAACAGACC<br>R: AAGTGCATCATCGTTGTTCATACA	IL-6 expression
IL-12 *p40*	F: AAGGAACAGTGGGTGTCCAG<br>R: CATCTTCTTCAGGCGTGTCA	IL-12β expression
IL-10	F: GGTTGCCAAGCCTTATCGGA<br>R: ACCTGCTCCACTGCCTTGCT	IL-10 expression

References:

1) Indukuri, V. (2013). Transposon based mutagenesis and mapping of transposon insertion sites within the Ehrlichia chaffeensis genome using semi random two-step PCR. Master of Science Thesis, Kansas State University.
2) Prima, V., Kaliberova, L.N., Kaliberov, S., Curiel, D.T., and Kusmartsev, S. (2017). COX2/mPGES1/PGE(2) pathway regulates PD-L1 expression in tumor-associated macrophages and myeloid-derived suppressor cells. *Proceedings of the National Academy of Sciences of the United States of America* 114**,** 1117-1122.
3) Niu, H., Rikihisa, Y., Yamaguchi, M., and Ohashi, N. (2006). Differential expression of VirB9 and VirB6 during the life cycle of *Anaplasma phagocytophilum* in human leucocytes is associated with differential binding and avoidance of lysosome pathway. *Cell Microbiol* 8**,** 523-534.

4) Miura, K., and Rikihisa, Y. (2009). Liver transcriptome profiles associated with strain-specific *Ehrlichia chaffeensis*-induced hepatitis in SCID mice. *Infect Immun* 77**,** 245-254.
