# Supplementary material for: Discovery of in vivo Virulence Genes of Obligatory Intracellular Bacteria by Random Mutagenesis
Source: Front Cell Infect Microbiol. 2020 Feb 4;10:2. doi: 10.3389/fcimb.2020.00002 (PMC7010607; doi:10.3389/fcimb.2020.00002)
Supplement: Supplementary file 2 [file Table_2.pdf]

## SUPPLEMENTARY TABLES

### Table S2. Himar insertion sites in *Ehrlichia* sp. HF genome

| Isolate ID | Genomic Insertion Loci | Locus ID | Gene Product | Homologies in Rickettsiales |
|---|---|---|---|---|
| H5 | 98810 | Intergenic | n/a | n/a |
| H19 | 245409 | EHF_0231 | Hypothetical protein | 32% Eucl, 28% Emu |
| H34 | 580467 | EHF_0522 | RmuC family protein | 96% Emu, 94% Eucl, 94% Emu, 89% Ech, 85% Eca, 85% Emin, 60% Ana |
| H35 | 364974 | Intergenic | n/a | n/a |
| H43A | 835933 | EHF_0733 | Hypothetical protein with GTA TIM-barrel-like domain protein | 94% Emu, 94% Eucl, 83% Ech, 79% Eca, 77% Emin, 63% Eru, 40% Wol, 38% Ana |
| H43B | 1050791 | EHF_RS04100 | Conserved hypothetical protein with SSL domain | 62% Emu, 44% Ech, 37% Emin, 34% Eca, 25% Eru |
| H43F | 133819 | EHF_0135 | trbC/VIRB2 family protein | 90% Emu, 84% Eucl, 78% Ech, 74% Emin, 71% Eru, 74% Ana |
| H47 | 839819 | Intergenic, 9 bp upstream of EHF_0735 | Major facilitator superfamily protein | 90% Emu, 90% Eucl, 83% Ech, 78% Eca, 76% Emin, 67% Eru, 36% Wol |
| H53C | 252371 | Intergenic, 343 up EHF_0242 | Conserved hypothetical protein | n/a |
| H53D-1 | 371562 | Intergenic | n/a | n/a |
| H53D-2 | 794748 | EHF_0703 | thiamine biosynthesis protein ThiC | 95% Emu, 95% Eucl, 92%, Ech, 88% Eca, 88% Emin, 88% Eru, 75% Nlo, 73% Aov, 72% Ama, 69% Aph |
| H53E | 150136 | EHF_0140 | Conserved hypothetical protein | 72% Emu, 50% Ech, 46% Emin, 48% Eca, 41% Eru, 25% Ana |
| H53F | 884488 | Intergenic | n/a | n/a |
| H54 | 241428 | Intergenic | n/a | n/a |
| H55 | 406316 | EHF_0382 | comEC/Rec2-related domain protein | 93% Emu, 93% Eucl, 80% Ech, 74% Eca, 73% Emin, 63% Eru, 47% Nlo, 45% Wol, 42% Aov, 39% Ama, 40% Aph |
| H55E | 973392 | Intergenic, 376 bp up EHF_0841 | COQ9 family protein | n/a |
| H56A | 1143100 | Intergenic, 157 bp up EHF_0993 and 126 bp up EHF_RS04480 | 120 kDa immunodominant surface protein, Ferredoxin | n/a |
| H56B | 353657 | EHF_0324 | lipoate-protein ligase B (LipB) | 95% Eucl, 95% Emu, 86% Ech, 80% Eca, 80% Emin, |

| | | | | |
|---|---|---|---|---|
| | | | | 76% Eru, 60% Aph, 57% Wol, 59% Nlo |
| H56D | 424981 | Intergenic, 130 bp up EHF_0399 and 439 bp up EHF_0402 | Polyprenyl synthetase protein, Ribonucleoside-diphosphate reductase subunit | n/a |
| H57A | 695771 | EHF_0623 | Conserved hypothetical protein | 47% Eucl, 46% Emu, 31% Ech, 24% Emin |
| H57B | 1122448 | Intergenic | n/a | n/a |
| H58A | 908826 | Intergenic | n/a | n/a |
| H58C | 871479 | EHF_0758 | Hypothetical protein | 43% Eucl, 44% Emu, 30% Eca, 30% Emin, 30% Ech |
| H58D | 163171 | Intergenic, 184 bp up EHF_0151 | type I secretion outer membrane/TolC family protein | n/a |
| H59 | 1106870 | EHF_0962 | Conserved hypothetical protein | 80% Emu, 80% Ech, 71% Eca, 62% Emin, 48% Eru |
| H60D | 144692 | Intergenic, 506 bp up EHF_0139 | Conserved hypothetical protein | n/a |
| H60E | 1142873 | EHF_0993 | 120 kDa immunodominant surface protein | 44% Emu, 38% Eucl, 41% Eca, 30% Ech |
| H62 | 1048515 | Intergenic | n/a | n/a |
| H64A | 163135 | Intergenic, 148 bp up EHF_0151 and 411 bp up EHF_0152 | type I secretion outer membrane/TolC family protein, ribosomal protein L13 | n/a |
| H64C | 341553 | Intergenic | n/a | n/a |
| H64F-1 | 253435 | EHF_0242 | Hypothetical protein | 78% Eucl, 78% Emu, 72% Ech, 51% Eca, 32% Eru |
| H64F-2 | 754040 | EHF_0671 | Conserved hypothetical protein | 78% Eucl, 78% Emu, 72% Ech, 50% Eca, 31% Eru |
| H65A | 117218 | Intergenic | n/a | n/a |
| H65D | 1018235 | EHF_0880 | Conserved hypothetical protein | 72% Emu, 52% Eca, 55% Emin, 53% Ech |
| H66A | 1074892 | EHF_0933 | Conserved hypothetical protein | 92% Emu, 82% Eca, 80% Ech, 76% Emin, 77% Eru, 48% Anas, 54% Nlo, 44% Rick |
| H66C | 357439 | Intergenic, 289 bp up EHF_0326 and 78 bp up EHF_0328 | DNA / pantothenate metabolism flavoprotein family protein, class II fumarate hydratase | n/a |
| H66F | 364844 | EHF_0332 | Conserved hypothetical protein | 74% Emu, 73% Eucl, 58% Ech, 45% Eca, 44% Emin, 38% Eru |
| H67A | 309891 | Intergenic, 560 bp up EHF_0288 | Phosphoglycolate hydrolase | n/a |
| H67C | 61587 | EHF_0066 | major outer membrane protein OMP1-2 | 93% Emu, 90% Eucl, 65% Ech, 56% Eca, 56% Emin, 45% Eru |
| H67E | 1074820 | Intergenic, 311 bp up EHF_0932 | ATP synthase subunit delta | n/a |

| | | | | |
|---|---|---|---|---|
| H67F | 792889 | EHF_0702 | Smr domain-containing protein | 87%. Emu, 87% Eucl, 76% Ech, 66% Eca, 64% Emin |
| H68B | 593034 | Intergenic, 199 bp up EHF_0532 | gram-negative porin family protein | n/a |
| H68C-1 | 671520 | Intergenic | n/a | n/a |
| H68C-2 | 269259 | Intergenic, 254 bp up EHF_0253 | Conserved hypothetical protein | n/a |
| H68E | 1035030 | Intergenic, 468 bp up EHF_0893 | Hypothetical protein | n/a |
| H69C | 569620 | Intergenic, 272 bp up EHF_0515 | Conserved hypothetical protein | n/a |
| H69E | 810865 | EHF_0717 | major facilitator family transporter | 96% Eucl, 94% Emu, 83% Ech, 74% Eca, 72% Emin, 68% Eru |
| H69F-1 | 579965 | EHF_0522 | DNA recombination protein RmuC | See H34 |
| H69F-2 | 45987 | EHF_0048 | major outer membrane protein OMP1-17 | 94% Emu, 93% Eucl, 85% Ech, 79% Emin, 70% Eca, 68% Eru, 67% Eew |
| H72A-1 | 161141 | EHF_0150 | DUF2497 domain-containing protein | 96% Eucl, 94% Emu, 85% Ech, 79% Eca, 78% Emin, 63% Eru, 35% Nlo, 30% Ana |
| H72A-2 | 1083158 | 307 bp up EHF_0939 | citrate (Si)-synthase | n/a |
| H72B | 1059627 | EHF_0919 | amidophosphoribosyltransferase ComF | 93% Emu, 82% Ech, 80% Eca, 74% Eru, 54% Wol, 55% Nlo, 45% Aph, 41% Ama, 39% Aov |
| H72C | 872359 | EHF_0758 | Hypothetical protein | See H58C |
| H72D | 63069 | Intergenic, 23 bp upstream of EHF_0067 | major outer membrane protein OMP1-1 | 89% Emu, 88% Eucl, 80% Ech, 72% Eca, 71% Emin, 66% Eew, 60% Eru, 43% Nlo |
| H72E | 248268 | EHF_0237 | DNA-3-methyladenine glycosylase | 93% Emu, 87% Ech, 80% Eca, 78% Emin, 73% Eru, 63% Wol, 63% Nlo, 56% Ots |
| H73A-1 | 72763 | EHF_0075 | dethiobiotin synthase | 91% Eucl, 92% Emu, 79% Ech, 72% Eca, 73% Emin, 70% Eru, 50% Ace, 50% Ama, 48% Aph |
| H73A-2 | 186084 | Intergenic, 516 bp up EHF_0173 | transcription termination factor Rho | n/a |
| H73B | 308181 | Intergenic, 390 bp up EHF_0286 | pyruvate, phosphate dikinase | n/a |
| H73C | 456831 | Intergenic | n/a | n/a |
| H73D-1 | 21696 | Intergenic | n/a | n/a |
| H73D-2 | 566454 | EHF_0513 | tellurium resistance protein TerC | 91% Emu, 90% Eucl, 83% Ech, 82% Eca, 79% Emin, 72% Eru, 52% Nlo, 52% Wol, 44% Aov, 44% Ama, 43% Ace |

| | | | | |
|---|---|---|---|---|
| H73E-1 | 490405 | EHF_0445 | trbL/VirB6 plasmid conjugal transfer family protein | 77% Emu, 73% Eucl, 82% Ech, 51% Eca, 52% Emin, 70% Eru |
| H73E-2 | 1016610 | EHF_0877 | transcriptional regulator NrdR | 99% Emu, 94% Ech, 89% Eca, 91% Emin, 88% Eru, 68% Nlo, 59% Ace, 58% Aov, 59% Aph |
| H73F | 456831 | Intergenic | n/a | n/a |
| H74A-1 | 47534 | Intergenic, 188 bp up EHF_0050 | P44/Msp2 family outer membrane protein | n/a |
| H74A-2 | 699943 | Intergenic, 68 bp up EHF_0625 and 363 bp up EHF_0626 | bifunctional FolC family protein, aspartyl/glutamyl-tRNA amidotransferase subunitA | n/a |
| H74B-1 | 366003 | EHF_0333 | dihydropteroate synthase | 94% Emu, 94% Eucl, 71% Eru, 56% Ech, 56% Eca, 56% Emin |
| H74B-2 | 822691 | Intergenic, 34 bp up EHF_0723 and 140 bp up EHF_0724 | DNA mismatch repair protein MutS, aspartyl/glutamyl-tRNA amidotransferase subunitA | EHF_0723:<br>97% Eucl, 98% Emu, 93% Ech, 88% Emin, 89% Eca, 79% Eru, 67% Nlo, 63% Wol, 59% Aph, 57% Ama, 57% Aov, 57% Ace, 51% Rick |
| H74C | 28140 | EHF_0031 | Chromosome partitioning ATPase ParA | 97% Emu, 98% Eucl, 89% Eru, 95% Ech, 94% Eca, 93% Emin, 89% Nlo, 70% Aov, 70% Ama, 67% Wol, 66% Rick |
| H74D | 1086516 | EHF_0943 | Hypothetical protein | 63% Eucl, 59% Emu, 37% Emin, 33% Ech, 36% Eca |
| H74E | 257823 | Intergenic | n/a | n/a |
| H74F-1 | 470413 | Intergenic, 16 bp up EHF_0435 | tRNA-Arg | n/a |
| H74F-2 | 886486 | EHF_0768 | conserved hypothetical protein | 61% Emu, 46% Ech, 32% Eru |
| H75A-1 | 354464 | Intergenic | n/a | n/a |
| H75A-2 | 428707 | Intergenic | n/a | n/a |
| H75A-3 | 188310 | Intergenic | n/a | n/a |
| H75B-1 | 45586 | EHF_0048 | major outer membrane protein OMP1-17 | See H69F-2 |
| H75B-2 | 1028718 | Intergenic, 319 bp up EHF_0885, 222 bp up EHF_0888 | NADP-dependent malic enzyme, ABC transporter | n/a |
| H75C | 308227 | EHF_0287 | RDD family protein | 92% Emu, 90% Eucl, 81% Ech, 81% Eca, 80% Emin, 67% Eru, 35% Ana, 42% Wol, 33% Rick |
| H75D-1 | 838124 | Intergenic | n/a | n/a |
| H75D-2 | 765049 | Intergenic, 19 bp up EHF_0677, | RNA pseudouridylate synthase family protein, extragenic suppressor protein suhB (suhB) | EHF_0677: 92% Emu, 91% Eucl, 79% Ech, 76% Eca, |

| | | | | |
|---|---|---|---|---|
| | | 502 bp up EHF_0678 | | 74% Emin, 67% Eru, 49% Wol, 47% Ana, 39% Rick |
| H75E | 939557 | Intergenic, 99 bp up EHF_0812 | patatin-like phospholipase family protein | n/a |
| H75F-1 | 869649 | EHF_0758 | hypothetical protein | See H58C |
| H75F-2 | 582106 | Intergenic | n/a | n/a |
| H75F-3 | 364700 | EHF_0332 | conserved hypothetical protein | See H66E |
| H75F-4 | 831375 | EHF_0732 | phage conserved hypothetical BR0599 family protein | 93% Emu, 83% Ech, 76% Emin, 77% Eca, 66% Eru, 49% Nlo, 40% Wol, 40% Ana |
| H76A-1 | 847362 | EHF_0743 | glutathione S-transferase | 97% Emu, 97% Eucl, 94% Ech, 94% Emin, 95% Eca, 86% Eru, 68% Nlo, 63% Wol, 60% Ana, 48% Rick |
| H76A-2 | 273766 | Intergenic | n/a | n/a |
| H76A-3 | 43980 | Intergenic, 122 bp up EHF_0045 | P44/Msp2 family outer membrane protein | n/a |
| H76B-1 | 1086497 | EHF_0943 | hypothetical protein | See H74D |
| H76B-2 | 517679 | EHF_0470 | conserved hypothetical protein | 79% Emu, 73% Ech, 66% Eca, 66% Emin, 50% Eru, 30% Ana |
| H76C | 1092639 | Intergenic, 262 bp up EHF_0950, 121 up EHF_0951 | protein translocase subunit SecF, archaeal holliday junction resolvase family protein | n/a |
| H76D-1 | 1112797 | Intergenic, 183 bp up EHF_0967 AND 844 bp up EHF_0966 | 1-acyl-sn-glycerol-3-phosphate acyltransferase | n/a |
| H76D-2 | 376020 | Intergenic, 142 bp up EHF_0341 | SPFH domain / Band 7 family (membrane) protein | n/a |
| H76E-1 | 150664 | EHF_0140 | conserved hypothetical protein | See H53E |
| H76E-2 | 149069 | Intergenic, 268 bp up EHF_0140 | conserved hypothetical protein | n/a |
| H76F | 179134 | EHF_0167 | aspartate kinase | 96% Emu, 95% Eucl, 93% Ech, 93% Eca, 91% Emin, 81% Eru, 65% Nlo, 51% Ana |
| H77A-1 | 792823 | EHF_0702 | smr domain protein | See H67F |
| H77A-2 | 765387 | Intergenic, 360 bp up EHF_0677 | pseudouridine synthase | n/a |
| H77A-3 | 41666 | Intergenic, 166 bp up EHF_0042 | P44/Msp2 family outer membrane protein | n/a |
| H77A-4 | 43288 | Intergenic | major outer membrane protein OMP1-19 | |
| H77A-5 | 150664 | EHF_0140 | conserved hypothetical protein | See H53E |
| H77B-1 | 43813 | EHF_0045 | major outer membrane protein OMP1-19 | See H77A-4 |
| H77B-2 | 516921 | Intergenic, 66 bp up EHF_0470 | conserved hypothetical protein | n/a |

| | | | | |
|---|---|---|---|---|
| H77B-3 | 46141 | EHF_0048 | major outer membrane protein OMP1-17 | See H69F-2 |
| H77C-1 | 516769 | Intergenic, 218 bp up EHF_0470 | conserved hypothetical protein | n/a |
| H77C-2 | 217716 | Intergenic | n/a | n/a |
| H77D-1 | 1125230 | Intergenic, 143 bp up EHF_0978 and 62 bp up EHF_0979 | 2,3,4,5-tetrahydropyridine-2,6-dicarboxylate N-succinyltransferase, hypothetical protein with MJ0042 family finger-like domain protein | n/a |
| H77D-2 | 525060 | Intergenic | n/a | n/a |
| H77E-1 | 448957 | Intergenic, 626 bp 425 bp up EHF_0423 | Xaa-Pro aminopeptidase | n/a |
| H77E-2 | 753371 | EHF_0671 | Hypothetical protein | See H64F-2 |
| H77F | 936950 | EHF_0810 | BolA protein | 83% Eucl, 94% Emu, 72% Ech, 73% Emin, 70% Eca, 64% Eru, 51% Wol, 40% Aph |
| H78 | 257958 | Intergenic | n/a | n/a |
| H79A-1 | 1132802 | Intergenic, 440 bp up EHF_0987 | GTP cyclohydrolase | n/a |
| H79A-2 | 813074 | EHF_0718 | Major Facilitator Superfamily (MFS) transporter | 94% Eucl, 95% Emu, 75% Ech, 75% Eca, 65% Eru, 37% Nlo, 35% Ana |
| H79A-3 | 64253 | EHF_0069 | nucleoside-diphosphate kinase | 96% Eucl, 97% Emu, 94% Ech, 91% Eca, 88% Eru, 73% Nlo, 64% Ana, 69% Wol, 64% Nhe, 64% Nsen |
| H79C-1 | 870872 | EHF_0758 | hypothetical protein | See H58C |
| H79C-2 | 834447 | EHF_0733 | hypothetical protein with GTA TIM-barrel-like domain protein | See H43A |
| H79C-3 | 152197 | Intergenic | | n/a |
| H79C-4 | 62134 | Intergenic, 438 bp up EHF_0066 | P44/Msp2 family outer membrane protein | n/a |
| H79D-1 | 513076 | EHF_RS02075 | Hypothetical protein (pseudogene due to frameshift) | n/a |
| H79D-2 | 827624 | Intergenic | n/a | n/a |
| H79E-1 | 946167 | Intergenic | n/a | n/a |
| H79E-2 | 407501 | EHF_0383 | sodium:alanine symporter family protein | 95% Eucl, 96% Emu, 90% Ech, 88% Emin, 87% Eca, 70% Eru, 60% Nlo, 57% Wol, 54% Ana, 39% Nhe, 37% Nri |
| H80A-1 | 42923 | Intergenic, 246 bp up EHF_0044 | major outer membrane protein OMP1-20 | n/a |
| H80A-2 | 1061828 | Intergenic, 83 bp up EHF_0922 | n/a | n/a |
| H80B-1 | 1044678 | EHF_0905 | hypothetical protein | 55 Eucl, 55% Emu, 36% Ech |

| | | | | |
|---|---|---|---|---|
| H80B-2 | 1005383 | Intergenic, 164 bp up EHF_0868 | n/a | n/a |
| H80B-3 | 112044 | EHF_0115 | Gamma carbonic anhydrase family protein | 92% Eucl, 95% Emu, 96% Ech, 80% Emin, 82% Eca, 64% Eru, 64% Nlo, 63% Wol, 63% Ana, 57% Nsen, 55% Nri |
| H80B-4 | 820620 | EHF_0723 | DNA mismatch repair protein MutS | See H74B-2 |
| H80B-5 | 1008450 | Intergenic | n/a | n/a |
| H80C-1 | 919082 | 986 bp up EHF_0795 | n/a | n/a |
| H80C-2 | 1131409 | EHF_0984 | conserved hypothetical protein | 88% Eucl, 85% Emu, 82% Ech72% Emin, 71% Eca, 62% Eru |
| H80C-3 | 382057 | EHF_0346 | cell division ZapA family protein | 96% Eucl, 98% Emu, 88% Ech, 80% Emin, 80% Eca, 76% Eru, 40-50% Wo, 35% Aov, 33% Ama |
| H80D-1 | 763703 | Intergenic | n/a | n/a |
| H80D-2 | 607098 | 139 bp up EHF_0545 | conserved hypothetical protein - pseudogene | n/a |
| H80D-3 | 810577 | EHF_0717 | Major Facilitator Superfamily (MFS) transporter | See H69E |
| H80E | 1018051 | 7 bp up EHF_0880 | conserved hypothetical protein | 79% Emu, 52% Eca, 55% Emin, 53% Ech |
| H80F | 929685 | 8 bp up EHF_0806 | 3-phosphoshikimate 1-carboxyvinyltransferase | 91% Eucl, 95% Emu, 74% Ech, 67% Emin, 67% Eca, 54% Eru, 37% Nlo |
| H81A-1 | 751268 | Intergenic | n/a | n/a |
| H81A-2 | 897331 | EHF_0775 | putative membrane protein | 65% Eucl, 69% Emu, 51% Ech, 53% Emin, 52% Eca, 48% Eru, 25% Ana |
| H81B-1 | 545195 | EHF_0497 | hypothetical protein | 64% Emu, 36% Eucl, 30% Ech, 23% Emin |
| H81B-2 | 920774 | 182 bp up EHF_0796, 546 bp up EHF_0797 | conserved hypothetical protein, tRNA-Leu | n/a |
| H81C-1 | 1013847 | Intergenic, 160 bp up EHF_0874 and 109 bp up EHF_0875 | hypothetical protein, conserved hypothetical protein | n/a |
| H81C-2 | 122477 | EHF_0125 | peptidase M16 family protein | 93% Emu, 89% Ech, 85% Emin, 85% Eca, 68% Eru, 50% Nlo, 43% Aph, 40% Ana, 43% Wol |
| H81D | 573935 | EHF_0515 | Chromosome segregation protein SMC (structural maintenance of chromosomes) archaeal type | 56% Eucl, 56% Emu, 44% Ech, 34% Emin |
| H81E | 1070542 | Intergenic | n/a | n/a |
| H82A-1 | 448861 | 722 bp up EHF_0423 | n/a | n/a |

| | | | | |
|---|---|---|---|---|
| H82A-2 | 161191 | EHF_0150 | conserved hypothetical protein | 96% Eucl, 94% Emu, 85% Ech, 79% Eca, 78% Emin, 63% Eru, 35% Nlo, 30% Ace |
| H82A-3 | 1098537 | EHF_0956 | octaprenyl-diphosphate synthase | 98% Eucl, 96% Emu, 88% Ech, 87% Eca, 86% Emin, 74% Eru, 62% Nlo, 52% Ace, 53% Ama, 51% Aov, 50% Aph, 52% Wol |
| H82B-1 | 509073 | 343 bp up EHF_0462 | n/a | n/a |
| H82B-2 | 45484 | EHF_0048 | major outer membrane protein OMP1-17 | See H69F-2 |
| H82C | 491800 | EHF_0446 | DUF2460 domain-containing protein | 95% Eucl, 95% Emu, 86% Ech, 83% Eca, 82% Emin, 76% Eru, 64% Nlo, 58% Wol, 49% Aph |
| H82D-1 | 737087 | Intergenic, 87 bp up EHF_0656 | bifunctional ADP-dependent NAD(P)H-hydratedehydratase/ NAD(P)H-hydrate epimerase | n/a |
| H82D-2 | 892485 | EHF_0772 | DNA mismatch repair protein MutL | 93% Eucl, 92% Emu, 81% Ech, 76% Eca, 77% Emin, 66% Eru, 59% Nlo, 57% Wol, 51% Aph, 40% Ots |
| H82E-1 | 188165 | Intergenic | n/a | n/a |
| H82E-2 | 753814 | EHF_0671 | Hypothetical protein | See H64F-2 |
| H82E-3 | 999937 | 279 bp up EHF_0861 | n/a | n/a |

**Abbreviations**:

Up: upstream of ORFs; Ech: *E. chaffeensis*; Eca: *E. canis*; Emu: *E. muris*; Emin: *E. minasensis*; Eru: *E. ruminantium*; Eucl: *E. muris* subsp. *Eauclairensis*; Eew: *E. ewingii*; Nhe: *Neorickettsia helminthoeca*; Nri: *N. risticii*; Nlo: Candidatus *Neoehrlichia lotoris*; Nsen: *N. sennetsu*, Ana: several *Anaplasma* species; Aph: *A. phagocytophilum*; Ace: *A. centrale*; Aov: *A. ovis*; Ama: *A. marginale*; Wol: *Wolbachia*; Ots: *Orientia tsutsugamushi*; Rick: *Rickettsia* species
